# Bayesian graphical models for computational network biology

**DOI:** 10.1186/s12859-018-2063-z

**Published:** 2018-03-21

**Authors:** Yang Ni, Peter Müller, Lin Wei, Yuan Ji

**Affiliations:** 1 Department of Statistics and Data Sciences, The University of Texas at Austin, Austin, 78712 TX USA; 2 Department of Mathematics, The University of Texas at Austin, Austin, 78712 TX USA; 3 NorthShore University HealthSystem, Evanston, 60201 IL USA; 4 Department of Public Health Sciences, The University of Chicago, Chicago, 60637 IL USA

**Keywords:** Directed graph, Undirected graph, Chain graph, Reciprocal graph, Causality

## Abstract

**Background:**

Computational network biology is an emerging interdisciplinary research area. Among many other network approaches, probabilistic graphical models provide a comprehensive probabilistic characterization of interaction patterns between molecules and the associated uncertainties.

**Results:**

In this article, we first review graphical models, including directed, undirected, and reciprocal graphs (RG), with an emphasis on the RG models that are curiously under-utilized in biostatistics and bioinformatics literature. RG’s strictly contain chain graphs as a special case and are suitable to model reciprocal causality such as feedback mechanism in molecular networks. We then extend the RG approach to modeling molecular networks by integrating DNA-, RNA- and protein-level data. We apply the extended RG method to The Cancer Genome Atlas multi-platform ovarian cancer data and reveal several interesting findings.

**Conclusions:**

This study aims to review the basics of different probabilistic graphical models as well as recent development in RG approaches for network modeling. The extension presented in this paper provides a principled and efficient way of integrating DNA copy number, DNA methylation, mRNA gene expression and protein expression.

## Background

This article starts with a comprehensive review of graphical models and recent development in constructing biological networks using reciprocal graphs (RG’s). In order to integrate multi-omics data including proteomics, we extend the work in [1] utilizing fundamental biological knowledge and the factorization of the joint distribution.

Computational network biology (CNB) is an emerging research field that encompasses theory and applications of network models to systematically study different molecules (DNA, RNA, proteins, metabolites and small molecules) and their complex interactions in living cells. CNB provides new insights in diverse research areas including system biology, molecular biology, genetics, pharmacology and precision medicine. The development of high-throughput profiling technologies allows for the interrogation of the status of many molecules in a cell at the same time, which makes it possible to jointly model these cell components with network modeling approaches. CNB is an interdisciplinary research area and has received contributions from researchers with various background. In this paper, we focus on network modeling approaches from a statistical perspective, namely, probabilistic graphical models (PGM) which allow a complete probabilistic description of interaction patterns between molecules and the associated uncertainties when estimating such interactions from noisy genomic and/or proteomic data.

PGM are probabilistic models for multivariate random variables whose conditional independence (also known as Markov property) structure is characterized by an underlying graph. PGM provides a concise, complete and explicit representation of joint distribution and allows for convenient Gibbs factorization of the density (if it exists) and hence local computations. The Markov property can be directly read off the graph through the notion of graph separation. PGM is closely related to causal inference, path analysis and expert system and finds a wide range of applications in political sciences, economics, genetics, biology, physics and psychology.

**Fig. 1:**
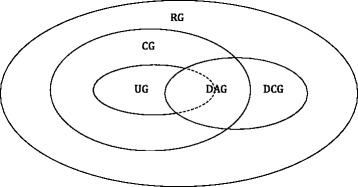
Venn’s diagram of different graphs. UG: undirected graph. DAG: directed acyclic graph. DCG: directed cyclic graph. CG: chain graph. RG: reciprocal graph

The most commonly used graphs are undirected graphs (UG) and directed acyclic graphs (DAG). UG represents symmetric interactions between variables whereas DAG allows for asymmetric ones that can be potentially interpreted as cause-effect relationships. Both UG and DAG are a special case of a more general graphical model known as chain graphs (CG) [2, 3] which allow for both symmetric and asymmetric conditional independence relationships. Despite of the flexibility of CG, the directed relationships are still constrained to be (block-) recursive, that is, reciprocal causality is prohibited. However, cyclic causal relationship are fundamental and ubiquitous in science, including, for example, the law of supply and demand in economics or feedback mechanism in gene regulatory networks. Spirtes [4] and Koster [5], in their respective seminal work, independently proposed coherent non-recursive graphical models including directed cycles of which the joint distribution was previously thought ill-defined ([6], p. 72). Spirtes [4] developed directed cyclic graphical (DCG) models which include DAG’s as a special case, and [5] proposed an even more general class of graphical models, reciprocal graphical (RG) models, which include CG (hence UG and DAG) and DCG as special cases. The relationships between the aforementioned graphs are depicted as a Venn’s diagram in Fig. 1.

In this paper, we focus on RG’s due to their statistical generality, the capability of modeling feedback loops and their still underappreciated popularity in the biostatistics and bioinformatics literature. The rest of this paper is structured as follows. We first review the basics of UG, DAG and RG in this section. In the next section, we provide a comprehensive review of novel Bayesian approaches in modeling molecular networks using RG. Then, we extend the RG approach in [1] to modeling The Cancer Genome Atlas (TCGA) ovarian cancer data that are measured on three different levels: DNA, RNA and proteins. The extension is non-trivial: we factorize an RG into two separate RG’s (one for DNA and RNA and the other for RNA and protein) and make coherent joint inference by exploiting two known biochemical processes: DNA transcribes to RNA and RNA in turn translates to protein, the latter also allowing us to estimate the direction of the relationship between RNA and protein which informs the directionality of the regulatory relationships between proteins. To the best of our knowledge, we are among the first to reconstruct multi-omics functional networks that consider mass spectrometry proteomics data generated by the CPTAC.

*Overview of directed, undirected and reciprocal graphical models.* Graphical models are a class of statistical models for a set of random variables ***Z***=(*Z*_1_,…,*Z*_*p*_)^*T*^ that provide a graphic representation of conditional independence relationships among the variables. Using graphical models allow practitioners to simplify inferences, obtain parsimonious solutions via variable selection, enable local computations, and define a framework for causal inference.

In a graphical model, variables are represented by a set of nodes and their associated interactions are represented by edges. A missing edge usually represents the conditional independence between the corresponding pair of random variables conditional on a certain set of other variables. The conditioning set depends on the chosen type of graph. Here, we provide a minimal set of terminologies required to describe the Markov properties in later sections.

A graph $\mathcal {G} = (V, E)$ is defined by a set of nodes *V*={1,…,*p*} and a set of directed and undirected edges *E*=*E*^*d*^∪*E*^*u*^. For a pair of nodes *i*,*j*∈*V*, we denote an undirected edge by *i*−*j* or {*i*,*j*}∈*E*^*u*^ and a directed edge by *i*→*j* or (*i*,*j*)∈*E*^*d*^. We write *i*↦*j* if there exists a path from *i* to *j*. A *path* is an ordered sequence (*i*_0_,…,*i*_*K*_) of distinct nodes except possibly *i*_0_=*i*_*K*_ such that {*i*_*k*−1_,*i*_*k*_}∈*E* or (*i*_*k*−1_,*i*_*k*_)∈*E* for *k*=1,…,*K*. A *cycle* is then a path with *i*_0_=*i*_*K*_. A *path component* is a set of nodes that are all connected by an undirected path. The *boundary* of a node *i* is bd(*i*)={*j*∣{*j*,*i*}∈*E* or (*j*,*i*)∈*E*} and the *boundary* of a subset *A*⊆*V* is $\text {bd}(A)=\bigcup _{i\in A}\text {bd}(i)\backslash A$. A node *i* is said to be a *parent* of node *j*, if (*i*,*j*)∈*E*. The set of parents of *j* is denoted by *pa*(*j*). The *ancestors* of node *i* are *an*(*i*)={*i*}∪{*j*|*j*↦*i*}. The *descendants* of node *i*, denoted by *de*(*i*), are the nodes *i* such that there is a path from *i* to *j*. The *non-descendants* of *i* are *nd*(*i*)=*V*∖(*de*(*i*)∪{*i*}). For *A*⊆*V*, the induced *subgraph* by *A* is $\mathcal {G}_{A}=(A,E_{A})$ where *E*_*A*_=*E*∩(*A*×*A*). A subset *A*⊆*V* is *anterior* if *bd*(*A*)=*∅* and the minimal anterior set of *A* is denoted by *an*(*A*). Note that we use *an*() to denote both ancestors and minimal anterior set; it should not cause any confusion because in fact *an*({*i*})=*an*(*i*). Notice that (minimal) anterior set [5] is equivalent to (minimal) ancestral set in [7]. A graph is *complete* if all nodes are joined by an edge. A complete subgraph that is maximal with respect to the subset relation is called *clique*. A useful procedure in defining Markov properties of graphs with directed edges is *moralization*. The moralization is performed in two steps. Make the boundary of each path component complete by adding undirected edges and then replace all directed edges by undirected ones. We call the resulting undirected graph *moral* graph, denoted by $\mathcal {G}^{m}=(V,E^{m})$.

We say that graph $\mathcal {G}$ is a undirected graph (UG) if *E*=*E*^*u*^ contains only undirected edges. And graph $\mathcal {G}$ is said to be a directed acyclic graph (DAG) if *E*=*E*^*d*^ contains only directed edges and no cycles. A *reciprocal graph* (RG) is a graph $\mathcal {G}$ that can have both directed and undirected edges and cycles, with the only restriction being no directed edges between nodes in the same path component [5]. In the following, we provide a concise overview of each type of graph. More comprehensive and technical details of the contents can be found in [7] and [6].

### Undirected graphical models

Let *P* denote the joint distribution of ***Z***=***Z***_*V*_=(*Z*_1_,…,*Z*_*p*_)^*T*^. We say *P* has a *Gibbs factorization* (or simply factorization) with respect to a UG $\mathcal {G}$ if its density *f*(***Z***) can be written as

1 $$\begin{array}{@{}rcl@{}}  f(\boldsymbol{Z})=\prod_{C\in \mathcal{C}}\psi_{C}(\boldsymbol{Z}_{C}) \end{array} $$

where $\mathcal {C}$ is the collection of cliques of $\mathcal {G}$ and *ψ*_*C*_ is an arbitrary nonnegative function on the domain of ***Z***_*C*_ for $C\in \mathcal {C}$.

Not surprisingly, the factorization property of *P* is closely related to its Markov properties; in fact, the former implies the latter. The Markov property of a UG relies on a notion of graph *separation*. For three disjoint subsets *A,B,C*∈*V* of nodes, *A* is said to be *separated* from *B* by *C* if every path between *A* and *B* includes a node in *C*. The global Markov property of a UG is given in the following definition.

#### **Definition 1**

(Global Markov property) Given a UG $\mathcal {G}$, the joint distribution *P* of ***Z***_*V*_ is global Markov with respect to $\mathcal {G}$ if for any three disjoint subsets *A*, *B* and *C* of *V*, ***Z***_*A*_ is independent of ***Z***_*B*_ given ***Z***_*C*_, written as ***Z***_*A*_ ⊥ ⊥***Z***_*B*_∣***Z***_*C*_ whenever *C* separates *A* and *B*.

The global Markov property always implies the local Markov property.

#### **Definition 2**

(Local Markov property) Given a UG $\mathcal {G}$, the joint distribution *P* of ***Z***_*V*_ is local Markov with respect to $\mathcal {G}$ if for any node *i*∈*V*, *Z*_*i*_ ⊥ ⊥***Z***_*V*∖{*bd*(*i*),*i*}_∣***Z***_*bd*(*i*)_.

The local Markov property in turn always implies the pairwise Markov property.

#### **Definition 3**

(Pairwise Markov property) Given a UG $\mathcal {G}$, the joint distribution *P* of ***Z***_*V*_ is pairwise Markov with respect to $\mathcal {G}$ if for any pair of nodes *i*,*j*∈*V*, *Z*_*i*_ ⊥ ⊥*Z*_*j*_∣***Z***_*V*∖{*i*,*j*}_.

In summary, factorization in (1) ⇒ global Markov property ⇒ local Markov property ⇒ pairwise Markov property. A natural question to ask is: is the relation also true backwards? The answer is yes when *P* has a positive and continuous density, which is summarized in the following theorem, often referred to as Hammersley-Clifford theorem [8].

#### **Theorem 1**

(Hammersley-Clifford) Suppose a probability distribution *P* has a positive and continuous density *f*. Then *P* satisfies the pairwise Markov property with respect to a UG $\mathcal {G}$ if and only if *f* factorizes according to $\mathcal {G}$.

When *P*=*N*(0,***Λ***^−1^) is multivariate Gaussian with precision matrix ***Λ***, undirected graphical models are defined by zero constraints on ***Λ***. There is a one-to-one correspondence between the graph structure $\mathcal {G}$ and the zero patterns in ***Λ***. Specifically, the precision matrix ***Λ*** is constrained to the cone of positive definite matrices with off-diagonal entry *Λ*_*ij*_=0 if and only if there is a missing edge in $\mathcal {G}$ between nodes *i* and *j*. Therefore, in the Gaussian case, learning the graph structure is equivalent to recovering the zero patterns in ***Λ***. Interested readers are referred to recent Bayesian approaches [9–11] and non-Bayesian approaches [12, 13].

### Directed graphical models

We say the distribution *P* factorizes with respect to a DAG $\mathcal {G}$ if its density *f*(***Z***) can be written as

2 $$  f(\boldsymbol{Z}) = \prod_{i = 1}^{p} f\left(Z_{i}\mid \boldsymbol{Z}_{pa(i)}\right).  $$

Similar to UG’s, the Markov property of a DAG can be also defined by the concept of graph separation. However, unlike UG’s, the separation is more complicated in DAG’s. There are two different but equivalent approaches in describing separation in a DAG. Pearl [14] introduced the notion of d-separation characterized by several conditions and then defined the global Markov property of a DAG in the same way as that of an UG except that separation is replaced by d-separation. Another approach [3] is based on the moralization and graph separation in UG’s. We elaborate more on the latter because it will be used again in defining Markov properties of RG’s. Similar to the previous section, we introduce global, local and pairwise Markov properties of DAG and then state the relationships between them. Recall that $\mathcal {G}^{m}$ denotes the moralized graph.

#### **Definition 4**

(Directed global Markov property) Given a DAG $\mathcal {G}$, the joint distribution *P* of ***Z***_*V*_ is global Markov with respect to $\mathcal {G}$ if for any three disjoint subsets *A*, *B* and *C* of *V*, ***Z***_*A*_ is independent of ***Z***_*B*_ given ***Z***_*C*_, written as ***Z***_*A*_ ⊥ ⊥***Z***_*B*_∣***Z***_*C*_ whenever *C* separates *A* and *B* in $\mathcal {G}_{an(A\cup B\cup C)}^{m}$.

#### **Definition 5**

(Directed local Markov property) Given a DAG $\mathcal {G}$, the joint distribution *P* of ***Z***_*V*_ is local Markov with respect to $\mathcal {G}$ if for any node *i*∈*V*, *Z*_*i*_ ⊥ ⊥***Z***_*nd*(*i*)_∣***Z***_*pa*(*i*)_.

#### **Definition 6**

(Directed pairwise Markov property) Given a DAG $\mathcal {G}$, the joint distribution *P* of ***Z***_*V*_ is pairwise Markov with respect to $\mathcal {G}$ if for any pair of nodes *i*,*j*∈*V*, *Z*_*i*_ ⊥ ⊥*Z*_*j*_∣***Z***_*nd*(*i*)∖{*j*}_.

Similar to UG’s, it is easy to show that factorization in (2) ⇒ directed global Markov property ⇒ directed local Markov property ⇒ directed pairwise Markov property. In fact, directed global and local Markov properties are equivalent [15]. Moreover, a theorem similar to Hammersley-Clifford’s theorem holds for DAG’s as well.

#### **Theorem 2**

([7]) Suppose a probability distribution *P* has a continuous density *f*. Then *P* satisfies the local Markov property with respect to a DAG $\mathcal {G}$ if and only if *f* factorizes according to $\mathcal {G}$. Furthermore, if the density *f* is positive, local and pairwise Markov properties are equivalent.

In a Gaussian DAG, each conditional distribution in (2) is a linear model

$$\begin{array}{@{}rcl@{}} f\left(Z_{i} \mid \boldsymbol{Z}_{pa(i)}\right) = N(Z_{i} \mid \sum_{j \in pa(i)} \beta_{ij} Z_{j},\sigma_{i}^{2}), \end{array} $$

where *N*(·∣*μ*,*σ*^2^) is a Gaussian density with mean *μ* and variance *σ*^2^. Essentially, a DAG is decomposed as a system of recursive and independent linear regressions for which the graph structure inference can be performed using standard model selection technique.

However, there is an important caveat in using DAG’s for statistical inference. That is the the notion of *Markov equivalence* – different DAG’s may induce the same set of conditional independence relationships. For example, the graphs *i*←*j*→*k* and *i*←*j*←*k* induce the same conditional independence relationship, *i* ⊥ ⊥*k*∣*j*. Both are equivalent to the UG *i*−*j*−*k*. In fact, any perfect DAG (i.e. a DAG without configuration *i*→*j*←*k*, that is, all parents are married) is Markov equivalent to a decomposable UG (i.e. UG in which all cycles of length four or more have a chord, which is an edge that is not part of the cycle but connects two nodes of the cycle). Conversely, given an undirected decomposable graph, there exists a Markov-equivalent perfect DAG; see [7] or [16]. One approach to avoid repeatedly analyzing Markov equivalent DAG’s is adopting a prior ordering of the nodes. The ordering may be obtained from subjective prior knowledge such as a reference network [17] or more objectively from an ordering learning algorithm such as order-MCMC [18]. For other recent DAG approaches, see [19–21] or [22].

### Reciprocal graphical models, simultaneous equation models and path diagrams

We introduce the global Markov property for RG’s in the following definition and omit the local and pairwise Markov properties since they are not as practically useful as in UG and DAG.

#### **Definition 7**

(Reciprocal global Markov property) Given a RG $\mathcal {G}$, the joint distribution *P* of ***Z***_*V*_ is global Markov with respect to $\mathcal {G}$ if for any three disjoint subsets *A*, *B* and *C* of *V*, ***Z***_*A*_ is independent of ***Z***_*B*_ given ***Z***_*C*_, written as ***Z***_*A*_ ⊥ ⊥***Z***_*B*_∣***Z***_*C*_ whenever *C* separates *A* and *B* in $\mathcal {G}_{an(A\cup B\cup C)}^{m}$.

Similarly to UG and DAG, there is also a Hammersley-Clifford-type theorem for RG’s ([5], Theorem 3.4).

However, while it is a beautiful theoretical result on the equivalence between the global Markov property and the Gibbs factorization in RG, the latter is difficult to work with in practice due to its complex form. Fortunately, in the Gaussian case, there is an easy connection between RG and simultaneous equation models (SEMs). To describe this connection, let ***Z***=(***Y***,***X***) be divided into two sets of variables ***Y*** and ***X*** and consider an SEM,

3 $$\begin{array}{@{}rcl@{}} \boldsymbol{Y}=\boldsymbol{A}\boldsymbol{Y}+\boldsymbol{B}\boldsymbol{X}+\boldsymbol{E}  \end{array} $$

where we assume the following five conditions:

1. (***Y***,***X***)∼*N*(0,***Σ***);
2. ***E*** ⊥ ⊥***X***;
3. ***I***−***A*** is invertible;
4. *Cov*(***E***) is diagonal;
5. ***Ψ***=*Cov*(***X***) is block diagonal with full diagonal blocks.

To link an SEM to an RG, we draw a path diagram $\mathcal {G}=(V,E)$ of SEM by the following rules:

(i) define nodes *V*={1,…,*p*,*p*+1,…,*p*+*q*} which represent (***Y***,***X***)=(*Y*_1_,…,*Y*_*p*_,*X*_1_,…,*X*_*q*_);
(ii) draw directed edges *E*^*d*^={(*j*,*i*)∣*a*_*ij*_≠0 or *b*_*i*,*j*−*p*_≠0}; and
(iii) draw undirected edges *E*^*u*^{{*i*,*j*}∣***Ψ***_*i*−*p*,*j*−*p*_≠0}.

**Fig. 2:**
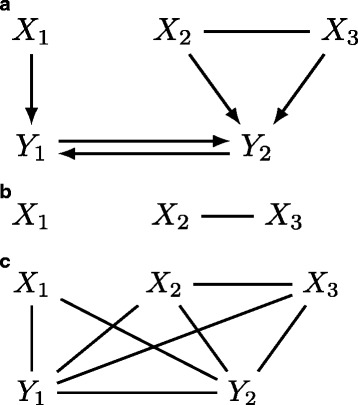
Illustration. **a** Path diagram $\mathcal {G}$ for SEM with configuration in (4). **b** Moral subgraph $\mathcal {G}_{\{X_{1},X_{2},X_{3}\}}^{m}$ induced by *an*(*X*_1_,*X*_2_,*X*_3_)={*X*_1_,*X*_2_,*X*_3_}. **c** Moral graph $\mathcal {G}^{m}$

In words, (i) we introduce a node for each variable in (***Y***,***X***) with nodes *j*=1,…,*p* corresponding to *Y*_*j*_ and *p*+*j* corresponding to *X*_*j*_ for *j*=1,…,*q*; (ii) nodes *i*=1,…,*p* (i.e. *Y*_*i*_ nodes) become targets of directed edges from node *j* if the corresponding *a*_*ij*_≠0 or *b*_*i*,*j*−*p*_≠0; (iii) we introduce undirected edges between *X*_*i*_ and *X*_*j*_ (i.e. nodes *i*+*p* and *j*+*p*) if ***Ψ***_*i*,*j*_≠0. Figure 2a shows an example of an RG with *p*=2 and *q*=3,

4 $$\begin{array}{@{}rcl@{}}  \boldsymbol{A}=\left[\begin{array}{cc}0&*\\ *&0\end{array}\right],~ \boldsymbol{B}=\left[\begin{array}{cccc}*&0&0\\0&*&*\end{array}\right] \text{and}~ \boldsymbol{\Psi}= \left[\begin{array}{ccc}*&0&0\\ 0&*&*\\0&*&*\end{array}\right], \end{array} $$

with ∗ indicating non-zero elements. With conditions C1-C5, the Markov properties of an SEM can be read off its path diagram.

#### **Theorem 3**

([5]) Let ***Y***=***AY***+***BX***+***E*** be an SEM satisfying conditions C1-C5. The joint distribution *P* of (***Y***,***X***) is global Markov with respect to the path diagram $\mathcal {G}$.

For example, the SEM with configuration in (4) implies *X*_1_ ⊥ ⊥*X*_2_,*X*_3_ which is evident by looking at the moral subgraph $\mathcal {G}_{an(X_{1},X_{2},X_{3})}^{m}=\mathcal {G}_{\{X_{1},X_{2},X_{3}\}}^{m}$ in Fig. 2b. Clearly, the empty set *∅* separates *X*_1_ and *X*_2_,*X*_3_. We can also find *X*_1_ ⊥ ⊥*X*_2_,*X*_3_∣*Y*_1_,*Y*_2_ by moralizing the entire path diagram (Fig. 2c) since set {*Y*_1_,*Y*_2_} separates *X*_1_ and *X*_2_,*X*_3_ in $\mathcal {G}^{m}$. However, we remark that the class of SEMs satisfying conditions C1-C5 is a subclass of RG. For instance, *i*→*j*−*k*←*l* is a CG (hence an RG) but does not correspond to an SEM. Similarly to DAG’s, DCG’s (or more generally RG’s) can also be divided into Markov equivalence classes; see [23–28] for characterization and learning of Markov equivalence classes of DCG’s.

## Methods

In this section, we will review recent RG approaches in modeling molecular networks including [29, 30] and [1].

### Modeling signaling pathways through latent variables

Telesca et al. [29] considered gene expressions *y*_*ij*_ for *p* genes measured over *n* samples, *i*=1,…,*n* and *j*=1,…,*p*. They follow [31] and introduce trinary latent variables *e*_*ij*_∈{−1,0,1} defining three possible categories of each expression *y*_*ij*_,

5 $$\begin{array}{@{}rcl@{}} e_{ij}=\left\{\begin{array}{ll}-1&\text{underexpressed gene } {j} \text{ in sample } {i}\\1&\text{overexpressed gene } {j} \text{ in sample } {i}\\0&\text{otherwise}\end{array}\right. \end{array} $$

The *e*_*ij*_ can be interpreted as an underlying biologic signal of under-, over- or normal expression. The motivation of the proposed inference is that only *e*_*ij*_ should be modeled without over-interpreting the additional noise in *y*_*ij*_ as biologically meaningful. Given *e*_*ij*_, a Gaussian-uniform mixture model is assumed for gene expression *y*_*ij*_,

6 $$\begin{array}{@{}rcl@{}}  \tilde{y}_{ij}|e_{ij}\sim f_{e_{ij},j}(\tilde{y}_{ij}), \end{array} $$

where $f_{-1,j}=U(-\kappa _{j}^{-},0),f_{1,j}=U\left (0,\kappa _{j}^{+}\right),f_{0,j}=N\left (0,\sigma _{j}^{2}\right)$ and $\tilde {y}_{ij}=y_{ij}-(\alpha _{i}+\mu _{j})$ is the normalized relative abundance, corrected for a sample-specific effect *α*_*i*_ and a gene-specific effect *μ*_*j*_. Conditional conjugate priors are assigned to $\alpha _{i},\mu _{j},\kappa _{j}^{-},\kappa _{j}^{+}$: $\alpha _{i}\sim N\left (0,\tau _{\alpha }^{2}\right), \mu _{j}\sim N\left (m_{\mu },\tau _{\mu }^{2}\right),\kappa _{j}^{-}\sim IG\left (a_{\kappa }^{-},b_{\kappa }^{-}\right),\kappa _{j}^{+}\sim IG\left (a_{\kappa }^{+},b_{\kappa }^{+}\right),\sigma _{j}^{2}\sim IG(a_{\sigma },b_{\sigma })$. Note that *α*_*i*_ and *μ*_*j*_ are not identifiable because *α*_*i*_+*μ*_*j*_=(*α*_*i*_−*c*)+(*μ*_*j*_+*c*) for any constant *c*. If desired, the identifiability problem can be resolved by setting $\sum _{i=1}^{n}\alpha _{i}=0$. For posterior simulation, the constraint can be implemented by setting *α*_*i*_←*α*_*i*_−*c* and *μ*_*j*_←*μ*_*j*_+*c* with $c=\sum _{i=1}^{n}\alpha _{i}$ at each iteration.

The dependence of gene expressions is modeled through the prior model for the latent variables *e*_*ij*_. In [29], they further introduced another set of latent Gaussian variables *z*_*ij*_ as probit scores for each trinary *e*_*ij*_,

$$e_{ij}=\left\{\begin{array}{cc} -1&\text{if \(z_{ij}>1\)}\\ 1&\text{if \(z_{ij}<-1\)}\\ 0&\text{otherwise.} \end{array}\right. $$

The continuous latent probit scores are then modeled by an SEM

$$z_{ij}=m_{ij}+\sum_{k\neq j}a_{jk}(z_{ik}-m_{ik})+\epsilon_{ij} $$

with $\epsilon _{ij}\sim N\left (0,s_{j}^{2}\right)$ and $m_{ij}=\boldsymbol {x}_{i}^{T}\beta _{j}$ for covariates ***x***_*i*_. Conjugate normal priors are assumed for regression coefficients *β*_*j*_ and *a*_*jk*_. By (3) this defines an RGM. Let ***A*** be the *p*×*p* matrix of which the diagonal elements are zeros and the off-diagonal (*j*,*k*) entries are *a*_*jk*_. Then the joint distribution of ***Z***_*i*_=(*z*_*i*1_,…,*z*_*ip*_)^*T*^ is given by

$$p(\boldsymbol{Z}_{i}|\boldsymbol{m}_{i},\boldsymbol{A})=N\left(\boldsymbol{m}_{i},(\boldsymbol{I}-\boldsymbol{A})^{-1}\boldsymbol{S}{(\boldsymbol{I}-\boldsymbol{A})^{-T}}\right), $$

where ***m***_*i*_=(*m*_*i*1_,…,*m*_*ip*_)^*T*^ and ***S***=*diag*(*s*_1_,…,*s*_*p*_). The structure of the path diagram $\mathcal {G}$ for this SEM is determined by the zero patterns in ***A***, i.e. *a*_*jk*_=0 if and only if there is a missing edge from *k* to *j*. They define the prior over graph $\mathcal {G}$ to be a subgraph of some fixed graph $\mathcal {G}_{0}=(V,E_{0})$. The idea is to specify $\mathcal {G}_{0}$ as some known maximum pathway. That is, $p(\mathcal {G}|\psi)=\psi ^{|E|}(1-\psi)^{|E_{0}|-|E|}$ where *ψ*∼*Beta*(*a*_*ψ*_,*b*_*ψ*_). Restricting $\mathcal {G}$ to subgraphs of $\mathcal {G}_{0}$ very effectively incorporates the prior information that might be available about an established biological pathway, using, for example, a public database such as KEGG. This restriction to subgraphs of $\mathcal {G}_{0}$ is important to mitigate nonidentifiability due to Markov equivalence between distinct graphs. Moreover, it also greatly reduces the graph search space, which allows for efficient posterior simulation through trans-dimensional Markov chain Monte Carlo (MCMC). Further discussion and detailed treatment of this topic can be found in [29].

The network structure in [29] is built on the latent Gaussian variables which are linked to the observed gene expressions through another layer of latent trinary variables. Therefore it defines an indirect dependence structure on the actual gene expression. [30] extend this work to allow for a more direct characterization of the dependence structure of the observed protein expressions. Particularly, they proposed a fully general dependence prior between latent binary variables and developed a parsimonious framework for Bayesian model determination that allows for local computation.

Telesca et al. [30] start with the same sampling model as in (5) and (6), but then deviate substantially from the earlier model by reducing the trinary variable *e*_*ij*_ to a binary indicator variable *z*_*ij*_=2*I*(*e*_*ij*_=1)−1. That is,

$${{} \begin{aligned} z_{ij}=\left\{ \begin{array}{cc} -1&\text{if protein } {j} \text{ is inactivated or neutral in sample } {i}\\ 1&\text{if protein } {j} \text{ is activated in sample } {i} \end{array}\right. \end{aligned}} $$

The motivation of this reduction is the simplicity of the prior model on the resulting binary vector ***Z***_*i*_=(*z*_*ij*_). In fact, it is possible [8] to describe all possible probability models *p*(***Z***_*i*_) that comply with a given conditional independence structure. See below. Also, investigators usually focus on protein activations in RPPA studies. The joint distribution of ***Z***_*i*_=(*z*_*i*1_,…,*z*_*ip*_)^*T*^ conditioned on the graph $\mathcal {G}$ is by an Ising model,

$$p(\boldsymbol{Z}_{i}|\mathcal{G},\boldsymbol{\alpha},\boldsymbol{\beta})\propto \exp\left(\sum_{j=1}^{p}\alpha_{j} z_{ij}+\sum_{\{j,k\}\in E^{m}}\beta_{jk}z_{ij}z_{ik}\right).$$

The model is completed with normal priors for *α*_*j*_ and *β*_*jk*_. In essence, the approach first maps an RG $\mathcal {G}=(V,E)$ to its moral graph $\mathcal {G}^{m}=(V,E^{m})$ and then defines the joint probability model for ***Z***_*i*_ based on $\mathcal {G}^{m}$. The prior model for the graph $\mathcal {G}$ is again hinged upon a known reference network $\mathcal {G}_{0}=(V,E_{0})$. However, it is not constrained to be a subgraph of the reference network as before. Instead, they assume $p(\mathcal {G}|\psi)\propto \psi ^{d(\mathcal {G},\mathcal {G}_{0})}$ with *ψ*∼*Beta*(*a*_*ψ*_,*b*_*ψ*_) where *d*(·,·) is a discrepancy measure. They choose $d(\cdot,\cdot)=|E^{c}\cap E_{0}|+\delta |E\cap E_{0}^{c}|$ with *δ*>1 which is a weighted sum of edges dropped from and added to the reference graph $\mathcal {G}_{0}$. With *δ*>1, the discrepancy measure allows for parsimonious inference by imposing a heavier penalty for adding edges than removing edges. A distinctive feature of this construction is that the prior decreases to zero exponentially fast as discrepancy measure increases, which allows the posterior simulation to be concentrated around the reference network. See [30] for more modeling and inference details of this topic.

### Integrative network analysis from multi-platform genomic data

Different types of molecules in a living cell do not act in isolation; in fact, DNA, RNA and proteins work closely together to carry out various functions for each cell. Studies that consider only one specific type of molecules remain unnecessarily restricted. In [1] they integrate DNA and RNA molecules for a more robust and biologically interpretable estimate of a gene network. In particular, they consider *p* gene expressions ***Y***=(*Y*_1_,…,*Y*_*p*_)^*T*^, together with corresponding copy number and methylation, collectively denoted by ***X***=(*X*_1_,…,*X*_2*p*_)^*T*^. They then exploit the central dogma of molecular biology that gene expression is produced by transcription from segments of DNA on which the copy number and methylation are measured, but the reverse processes are rare and biologically uninterpretable. The importance of this restriction is that in a network of ***Z***=(***Y***,***X***) some edges have fixed pre-determined directions corresponding this observation. This in turn allows us to report meaningful inference on the directions of the remaining edges. Similar idea but in a different context has been proposed by [32–34]. With this assumption, they then model the regulatory relationships between copy number, methylation and gene expression with the following SEM

7 $$\begin{array}{@{}rcl@{}} \boldsymbol{Y}=\boldsymbol{A}\boldsymbol{Y}+\boldsymbol{B}\boldsymbol{X}+\boldsymbol{E} \end{array} $$

where $\boldsymbol {A}=(a_{ij})\in \mathbb {R}^{p\times p}$ with zeros on the diagonal, $\boldsymbol {B}=(b_{ij})\in \mathbb {R}^{p\times 2p}$ and ***E***=(*ε*_1_,…,*ε*_*p*_)^*T*^∼N_*p*_(0,***Σ***). The SEM in (7) immediately prohibits gene expression from regulating copy number or methylation since there is no ***X*** on the right-hand side of the equation. It also explicitly allows for feedback loops between the genes which are quite common motifs in molecular networks and have key functional roles in many cellular processes such as regulating gene expressions and acting as bistable switches [35]. Such feature is missing in DAG’s, UG’s and CG’s.

Next they assign thresholded priors [36] for ***A*** and ***B***,

$$a_{ij}=\tilde{a}_{ij}\textrm{I}(\mid\tilde{a}_{ij}\mid>t_{i})\text{~~and~~}b_{ik}=\tilde{b}_{ik}\textrm{I}(\mid\tilde{b}_{ik}\mid>t_{i}), $$

for *i*=1,…,*p*, *j*≠*i* and *k*=2*i*−1,2*i*. The threshold parameter *t*_*i*_ controls the minimum effect sizes of *a*_*ij*_ and *b*_*ik*_ and is assigned a uniform hyperprior *t*_*i*_∼Uniform(0,*t*_0_). Normal priors are given to $\tilde {a}_{ij}$ and $\tilde {b}_{ik}$, $\tilde {a}_{ij}\sim \textrm {N}\left (0,\tau _{ij}^{(a)}\right)$ and $\tilde {b}_{ik}\sim \textrm {N}\left (0,\tau _{ik}^{(b)}\right)$ and conjugate hyperpriors $\tau _{ij}^{(a)}\sim \text {IG}(\alpha _{\tau },\beta _{\tau })$ and $\tau _{ik}^{(b)}\sim \text {IG}(\alpha _{\tau },\beta _{\tau })$. The thresholded priors enjoy nice theoretical properties as they are closely related to spike-and-slab prior and non-local prior as shown in [36–38]. We refer readers to [1] for a more comprehensive description of the work.

### Reverse engineering gene networks with heterogeneous samples

Genomic data are often heterogeneous in the sense that the samples can be naturally divided into *observed* or *hidden* groups. For example, in a pan-cancer study, patients are normally grouped by the known cancer types whereas when studying a specific cancer, patients can be split into homogeneous latent subtypes. The inferential goals of these two cases are typically quite different. In the case with observed groups, much of the statistical work has been devoted to building models that allow information to be shared across groups. Such approaches improve the statistical power in graph estimation especially for groups with very limited samples by borrowing strength from other groups. For hidden groups, the underlying task becomes a clustering problem which aims to partition subjects into homogeneous clusters. In this section, we introduce two hierarchical RG models that are suitable for heterogeneous datasets. Interested readers can find more details in [38].

Suppose there are *C* known groups in the data. Let {*s*_*i*_=*c*} be the group indicator for patient *i* in group *c*∈{1,…,*C*}. Denote ***Y***_*c*_={*Y*_*i*_; *s*_*i*_=*c*} and ***X***_*c*_={*X*_*i*_; *s*_*i*_=*c*} the set of observations for patients in group *c*. Then [38] entertain a group-specific SEM

$$\boldsymbol{Y}_{c}=\boldsymbol{A}_{c}\boldsymbol{Y}_{c}+\boldsymbol{B}_{c}\boldsymbol{X}_{c}+\boldsymbol{E}_{c} $$

where ***A***_*c*_ = (*a*_*c*,*ij*_),***B***_*c*_ = (*b*_*c*,*ij*_)and ***E***_*c*_ = (*ε*_*c*,1_,…,*ε*_*c*,*p*_)^*T*^ ∼ *N*_*p*_(0,***Σ***_*c*_) with $a_{c,ij}=\tilde {a}_{c,ij}\textrm {I}(\mid \tilde {a}_{c,ij}\mid >t_{i})$ and $b_{c,ik}=\tilde {b}_{c,ik}\textrm {I}(\mid \tilde {b}_{c,ik}\mid >t_{i})$. To link the graphs across groups, they impose multivariate normal priors on the edge strength

$$\begin{array}{@{}rcl@{}} &&\tilde{\boldsymbol{a}}_{ij}=\left(\tilde{a}_{1,ij},\dots,\tilde{a}_{C,ij}\right)^{T}\sim N_{C}\left(0,\tau_{ij}^{(a)}\boldsymbol{\Omega}\right),\\ &&\tilde{\boldsymbol{b}}_{ik}=\left(\tilde{b}_{1,ik},\dots,\tilde{b}_{C,ik}\right)^{T}\sim N_{C}\left(0,\tau_{ik}^{(b)}\boldsymbol{\Omega}\right). \end{array} $$

The covariance matrix $\boldsymbol {\Omega }\!\!\!\,=\!(\omega _{cc^{\prime }})$ connects edge strength and graph structure across groups. When $\omega _{cc^{\prime }}$ is close to ±1, $\tilde {a}_{c,ij}$ and $\tilde {a}_{c^{\prime },ij}$ tend to have similar absolute values, which gives rise to a high probability that the edge *i*←*j* is included or excluded in groups *c* and *c*^′^ simultaneously since the threshold *t*_*i*_ is shared across groups. Therefore, graphs from group *c* and *c*^′^ are more likely to share common edges. On the other hand, when $\omega _{cc^{\prime }}$ is close to 0, the association between groups *c* and *c*^′^ is negligible. They do not fix ***Ω***. Instead, they use an inverse-Wishart prior, ***Ω***∼*IW*(*ν*,***Ψ***) and let the data dictate how strong the associations should be.

The Bayesian hierarchical model above allows for easy extension to the case where the groups are unknown. Ni et al. [38] simply augment the model with a Dirichlet-multinomial (DM) allocation model on the group indicator *c*|***π***,*C*∼Multinomial(1,*π*_1_,…,*π*_*C*_) and ***π***|*C*∼Dir(*η*,…,*η*). The number of clusters *C* is usually unknown; they assume a geometric prior for *C*∼Geo(*ρ*), which eliminates the need to fix *C* a priori. They set the covariance matrix ***Ω*** to be diagonal since the goal of clustering is to split samples into groups with disparate networks rather than encourage similarity across the clusters. Alternatively, one can also set ***Ω*** to be a Stieltjes matrix (i.e. a positive definite matrix with nonpositive off-diagonal entries) to induce repulsive graphs.

## Results

In this section, we generalize the RG approach of [1] by incorporating protein expressions in modeling the molecular network. Proteins provide important and orthogonal information in reverse engineering the network because they represent the downstream cumulative effect of changes that happen at the DNA and RNA levels and are more directly related to the phenotypical changes in cancer cells. Clinical utilization of genomic data alone show limited benefits, which is partly due to the poor concordance between gene and protein expressions [39, 40]. Many factors such as complex interactions between different types of molecules are responsible for the discrepancy between gene and protein abundance. The goal of this analysis is to explicitly model such complex interactions. We choose RG because it allows for efficient computation and great interpretability and is suitable to model reciprocal causality such as feedback mechanism.

We use the same notations ***Y***=(*Y*_1_,…,*Y*_*p*_)^*T*^ and ***X***=(*X*_1_,…,*X*_2*p*_)^*T*^ to denote gene expressions and the matched copy number and methylation. Importantly, additionally, for each gene *Y*_*j*_, there is also the associated protein expression, denoted by *Z*_*j*_ for *j*=1,…,*p*. We extend the SEM in (7) to

8 $$  \begin{aligned} \boldsymbol{Y}&=\boldsymbol{A}\boldsymbol{Y}+\boldsymbol{B}\boldsymbol{X}+\boldsymbol{E_{Y}}\\ \boldsymbol{Z}&=\boldsymbol{C}\boldsymbol{Z}+\boldsymbol{D}\boldsymbol{Y}+\boldsymbol{E_{Z}} \end{aligned}  $$

where $\boldsymbol {A}=(a_{ij})\in \mathbb {R}^{p\times p}$ and $\boldsymbol {C}=(c_{ij})\in \mathbb {R}^{p\times p}$ have zeros on the diagonal, $\boldsymbol {B}=(b_{ij})\in \mathbb {R}^{p\times 2p}$, $\boldsymbol {D}=(d_{ij})\in \mathbb {R}^{p\times p}$ and independent errors ***E***_***Y***_∼N_*p*_(0,***Σ***_***Y***_) and ***E***_***Z***_∼N_*p*_(0,***Σ***_***Z***_) with diagonal covariance matrices ***Σ***_***Y***_=*diag*(*σ*_1_,…,*σ*_*p*_) and ***Σ***_***Z***_=*diag*(*λ*_1_,…,*λ*_*p*_). In essence, model (8) defines the following joint probability model for (***Y***,***Z***) given ***X***,

9 $$  {{} \begin{aligned} p(\boldsymbol{Y},\boldsymbol{Z}|\boldsymbol{X})&=p(\boldsymbol{Z}|\boldsymbol{Y})p(\boldsymbol{Y}|\boldsymbol{X}),\\ p(\boldsymbol{Y}|\boldsymbol{X})&= \textrm{N}_{p}\left\{(\boldsymbol{I}-\boldsymbol{A})^{-1}\boldsymbol{B}\boldsymbol{X},(\boldsymbol{I}-\boldsymbol{A})^{-1}\boldsymbol{\Sigma_{Y}}(\boldsymbol{I}-\boldsymbol{A})^{-T}\right\},\\ p(\boldsymbol{Z}|\boldsymbol{Y})&= \textrm{N}_{p}\left\{(\boldsymbol{I}-\boldsymbol{C})^{-1}\boldsymbol{D}\boldsymbol{Y},(\boldsymbol{I}-\boldsymbol{C})^{-1}\boldsymbol{\Sigma_{Z}}(\boldsymbol{I}-\boldsymbol{C})^{-T}\right\}. \end{aligned}}  $$

The network structure is embedded in the parameters ***A***,***B***,***C*** and ***D***. In this analysis, we restrict our attention to *cis-*regulatory relationships between DNAs and RNAs by constraining *b*_*ij*_ to 0 for *j*≠2*i*−1 or 2*i* for *i*=1,…,*p*. That is, copy number and methylation of gene *i* can only affect gene expression *i*. The interpretation of model (9) is given as follows: *b*_*i*,2*i*−1_≠0 if and only if copy number *i* is associated with its gene expression; *b*_*i*,2*i*_≠0 if and only if methylation *i* is associated with its gene expression; *a*_*ij*_≠0 if and only if gene *j* regulates gene *i*; *c*_*ij*_≠0 if and only if protein *j* regulates protein *j*; and *d*_*ij*_≠0 if and only if gene *j* regulates protein *i*. We use the thresholded prior for ***A***,***B***,***C*** and ***D***. For error variances, we assume inverse-gamma priors, *σ*_*j*_,*λ*_*j*_∼*IG*(*a*,*b*) for *j*=1,…,*p* which imply an inverse gamma conditional posterior distribution which in turn allows the use of a Gibbs sampling transition probability by sampling from the respective inverse gamma. The complete MCMC algorithm is provided in Algorithm 1.



**Fig. 3:**
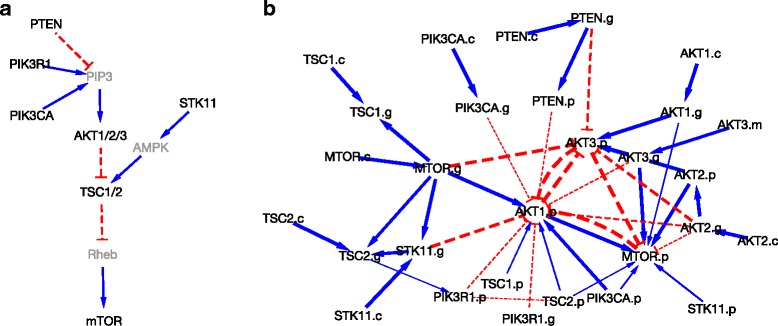
Genomic networks. **a** Reference network of core members of PI3K pathway. Molecules that are not used in our analysis are in gray. The blue solid lines with arrow heads are activations; red dashed lines with horizontal bars are inactivations. **b** Recovered network. The suffixes represent: c=copy number, m=methylation, g=gene and p=protein. Edge width is proportional to the posterior probability of inclusion. Disconnected molecules are not shown

Using TCGA-Assembler ([41], version 2), we acquired, processed and combined TCGA ovarian cancer data, including copy number, methylation and gene expression from the Genomic Data Commons (GDC) and mass spectrometry proteomic samples generated by the Clinical Proteomic Tumor Analysis Consortium (CPTAC). The resulting dataset contains 104 samples. In this case study, we focus on *p*=10 genes that are core members of the PI3K pathway [42], as shown in Fig. 3a, by playing a critical role in cell cycle progression, survival, motility, angiogenesis and immune surveillance [43]. It is one of the most frequently altered pathways in ovarian cancer [44, 45]. For each gene, we have matched copy number, methylation, gene expression and protein expression (i.e. 40 nodes in total). We report inference based on 100,000 MCMC samples (thinned out to every 10th iteration) after discarding the first 50,000 iterations as burn-in.

The recovered network is shown in Fig. 3b where the blue solid lines represent activation whereas the red dashed lines are inactivations. The edge width is proportional to the posterior probabilities of inclusion *p*(*a*_*ij*_≠0|***X***,***Y***,***Z***), *p*(*b*_*ij*_≠0|***X***,***Y***,***Z***), *p*(*c*_*ij*_≠0|***X***,***Y***,***Z***) and *p*(*d*_*ij*_≠0|***X***,***Y***,***Z***), which can be approximated by MCMC sample averages, for example, $p(a_{ij}\neq 0|\boldsymbol {X},\boldsymbol {Y},\boldsymbol {Z})=\frac {1}{L}\sum _{l=1}^{L}I\left (a_{ij}^{(l)}\neq 0\right)$ where the superscript (*l*) denotes the *l*th MCMC sample and *L* is the total number of samples. The suffixes represent four different type of molecules: c=copy number, m=methylation, g=gene and p=protein. For clarity we do not show disconnected molecules. The degree of each node in Fig. 3b is reported in Table 1. The top three highly connected nodes are the protein AKT1 which is involved in 15 regulatory activities, the protein mTOR with 10 activities and the protein AKT3 with 9 activities. AKT1 and AKT3 belong to the same AKT family which is known to have shown strong oncogenic function and is a key mediator of PI3K pathway function [46]. AKT isoforms are often phosphorylated in ovarian cancers and may play a role in mediating the progression of late-stage serous ovarian carcinomas [47]. mTOR plays a critical role in regulating cell growth and proliferation by integrating signals including growth factors, nutrients, energy and stress [48, 49]. We also capture the well-known positive regulatory relationship between AKT1 and its downstream target mTOR [45, 49]. Other findings that are consistent with the biological literature include the inhibitory relationship between proteins PTEN and AKT1 and stimulatory relationship between proteins PIK3CA and AKT1 [50, 51]. Interestingly, the recovered network includes a negative feedback from proteins of mTOR to AKT1. Recent studies [45, 52–54] confirmed that such feedback mechanism can be either positive or negative depending on many factors including cell types and conditions.

**Table 1:** Degrees of molecules from recovered network in Fig. 3b

Molecule	Degree	Molecule	Degree
AKT1.p	15	PTEN.p	2
mTOR.p	10	PIK3CA.p	2
AKT3.p	9	AKT1.c	1
mTOR.g	6	AKT2.c	1
AKT2.g	5	AKT3.m	1
AKT3.g	4	mTOR.c	1
STK11.g	4	PIK3CA.c	1
TSC2.g	4	PTEN.c	1
AKT1.g	3	STK11.c	1
PTEN.g	3	TSC1.c	1
AKT2.p	3	TSC2.c	1
PIK3R1.p	3	PIK3R1.g	1
TSC2.p	3	STK11.p	1
PIK3CA.g	2	TSC1.p	1
TSC1.g	2		

Inference includes some surprising findings. For example, PIK3R1 is expected to activate rather than inactivate AKT1. Although the unexpected result may simply be a false positive, it deserves further experimental validation.

## Discussion and conclusions

We have reviewed the basics of UG’s, DAG’s and RG’s and discussed recent RG-based approaches in modeling molecular networks. The extension of the approach in [1] allows to integrate multi-platform data including copy number, methylation, gene expression and protein expression. Our approach can be further be generalized to incorporating other data types such as single nucleotide polymorphisms (SNPs), mutation status and microRNAs. SEM-based RG approaches, however, cannot be directly applied to SNPs and mutation status because they have discrete support and the condition C1 requires the data to be multivariate normal. This limitation can be addressed by imputing latent normal variables. Although we do not find serious violation of normality assumption in our data, if desired, one could adopt the approach of [29] and introduce additional layers of hidden variables when the data are seemingly non-normal.

Another important assumption of the discussed approaches in this paper is homogeneity across samples. It is an unrealistic assumption in many applications, especially in oncology where the consensus is that tumors are extremely heterogeneous. Characterizing tumor heterogeneity can fundamentally improve our understanding of the cancer biology and practically allow us to divide heterogeneous patient population into homogeneous subpopulations so that refined personalized treatments can be developed targeting specific subgroups of cancer patients. Several approaches have been proposed for modeling networks with heterogeneous samples [38, 55–57].
